# Survival outcomes and safety of carmustine wafers in the treatment of high-grade gliomas: a meta-analysis

**DOI:** 10.1007/s11060-015-1724-2

**Published:** 2015-01-29

**Authors:** Sajeel A. Chowdhary, Timothy Ryken, Herbert B. Newton

**Affiliations:** 1 Department of Neuro-Oncology, Florida Hospital Cancer Institute, 2501 N. Orange Avenue, Suite 286, Orlando, FL 32804 USA; 2 Department of Neurosurgery, Iowa Spine and Brain Institute, 2710 St. Francis Drive, Waterloo, IA 50702 USA; 3 Departments of Neurology, Neurosurgery, and Oncology, Wexner Medical Center at the Ohio State University and James Cancer Hospital, M410-B Starling-Loving Hall, 320 West 10th Avenue, Columbus, OH 43210 USA

**Keywords:** BCNU, Carmustine, Gliadel, Glioblastoma, Glioma, High-grade, Meta-analysis, Wafer

## Abstract

Carmustine wafers (CW; Gliadel^®^ wafers) are approved to treat newly-diagnosed high-grade glioma (HGG) and recurrent glioblastoma. Widespread use has been limited for several reasons, including concern that their use may preclude enrollment in subsequent clinical trials due to uncertainty about confounding of results and potential toxicities. This meta-analysis estimated survival following treatment with CW for HGG. A literature search identified relevant studies. Overall survival (OS), median survival, and adverse events (AEs) were summarized. Analysis of variance evaluated effects of treatment (CW vs non-CW) and diagnosis (new vs recurrent) on median survival. The analysis included 62 publications, which reported data for 60 studies (CW: n = 3,162; non-CW: n = 1,736). For newly-diagnosed HGG, 1-year OS was 67 % with CW and 48 % without; 2-year OS was 26 and 15 %, respectively; median survival was 16.4 ± 21.6 months and 13.1 ± 29.9 months, respectively. For recurrent HGG, 1-year OS was 37 % with CW and 34 % without; 2-year OS was 15 and 12 %, respectively; median survival was 9.7 ± 20.9 months and 8.6 ± 22.6 months, respectively. Effects of treatment (longer median survival with CW than without; *P* = 0.043) and diagnosis (longer median survival for newly-diagnosed HGG than recurrent; *P* < 0.001) on median survival were significant, with no significant treatment-by-diagnosis interaction (*P* = 0.620). The most common AE associated with wafer removal was surgical site infection (SSI); the most common AEs for repeat surgery were mass effect, SSI, hydrocephalus, cysts in resection cavity, acute hematoma, wound healing complications, and brain necrosis. These data may be useful in the context of utilizing CW in HGG management, and in designing future clinical trials to allow CW-treated patients to participate in experimental protocols.

## Introduction

High-grade gliomas (HGG; WHO grade 3 or 4) account for the majority of newly-diagnosed malignant brain tumors, with glioblastoma multiforme (GBM) representing the most common subtype [1]. These highly infiltrative and aggressive tumors generally have a poor prognosis, as they are difficult to treat and recurrence is common [2]. Treatment for HGG generally includes surgical resection followed by radiotherapy and chemotherapy [2]. In particular, the addition of the alkylating agent temozolomide (TMZ) to post-surgical radiotherapy and as adjuvant therapy has become standard treatment for many patients with HGG [2, 3]. Factors associated with prolonged survival include complete resection (≥98 % of tumor volume) [4], younger age [5], better performance status [5], MGMT promoter status [6, 7], oligodendroglial phenotype [5], p53 mutation [8], and IDH1 mutation [9].

Carmustine wafer (CW) implant (Gliadel^®^ Wafer, Arbor Pharmaceuticals, LLC, Atlanta, GA) is approved for treatment of newly-diagnosed HGG as an adjunct to surgery and radiation and for treatment of recurrent GBM as an adjunct to surgery [10]. Local chemotherapy with CW was shown to significantly increase survival compared with placebo in newly-diagnosed HGG [11, 12] and in recurrent GBM [13]. Risks associated with CW include cerebral edema, healing abnormalities, intracranial infections, seizures, intracranial hypertension, and cerebrospinal fluid leaks [14].

Treatment guidelines recommend CW as appropriate for some patients (e.g., patients in whom near total resection is feasible [Category 2B recommendation] [2] or in whom craniotomy is indicated [Level II recommendation] [15]); however, questions remain as to its optimal use. For example, randomized controlled trials (RCT) comparing CW and TMZ as single treatments have not been conducted, and while several reports on the use of TMZ following CW implantation have been published (see review by Dixit et al. [16]), there remain concerns about the safety of this approach [2]. Currently, many clinical trials of new chemotherapies exclude patients treated with CW [2, 17] because of concerns about potential toxicities, confounding of results (e.g., due to wafer-induced imaging changes), and a paucity of reliable survival statistics. More reliable data regarding expected survival times with CW might be helpful in the context of designing future clinical trials, so that new protocols might accommodate the use of CW as part of a comprehensive approach utilizing multiple treatment modalities maximizing benefit to patients.

This meta-analysis was designed to estimate survival times for patients treated with CW for newly-diagnosed or recurrent HGG, using data from published studies.

## Methods

### Search strategy and study selection

A literature search was conducted in January, 2014 using Medline (includes PubMed), Embase, and BIOSIS, with the following search criteria: gliadel OR [(“BCNU” OR carmustine) AND (polymer OR polymers OR wafer* OR polifeprosan OR interstitial)] AND (glioma OR glioblastoma); no restrictions on publication date were used. The abstract of each publication was screened to determine relevance. Much of the published evidence on CW is derived from retrospective studies of heterogeneous populations and varying treatment regimens, which generally precludes inclusion of these publications in meta-analyses. However, in an effort to utilize as much of the available data as possible and increase the generalizability of our results, we chose to exclude only preclinical or phase 1 studies, individual case reports, or small case series (n < 10); also excluded were review articles, editorials, and studies of carmustine administered in a formulation other than wafers. Each remaining publication was reviewed to determine if overall survival or selected safety/toxicity outcomes (seizures, wound healing complications, infection, or mass effect) were reported for patients treated with CW. Relevant congress abstracts published in 2009 or later were identified (via the Northern Light database in addition to sources listed above) and screened using the same process and criteria described above. Abstracts of studies with full results published were also excluded.

### Data collection

Data were extracted and reviewed. The following were collected: (1) characteristics of study participants, including age, sex, diagnosis (new or recurrent), tumor grade (grade 3 or 4 vs grade 4 only); (2) study treatment (specific treatment regimens, general categories of CW alone, CW + other treatment(s), no CW, radiotherapy use); (3) survival outcomes (1-, 2-, and 3-year survival rate; median survival time); (4) safety outcomes (adverse events [AEs], deaths due to AEs, wafer removal, repeat surgery).

### Statistical analyses

Overall survival (OS) rates at 1, 2, and 3 years, and median survival time were summarized by tumor grade (grade 3 or 4 vs grade 4 only), by new or recurrent diagnosis, and by use of CW with or without TMZ. A factorial analysis of variance was performed to evaluate the effects of treatment (CW vs no CW), diagnosis (new vs recurrent), and use of TMZ (among CW-treated patients) on median survival. All statistical analyses including tests of hypotheses and *P*-values are based on weighted statistics, where the weights were derived from the total safety or efficacy sample size. Statistical significance for all weighted statistical tests were set at *P* ≤ 0.05. No Bonferroni correction for multiple inferences was applied. All analyses were performed using SAS (version 9.2). The incidence of safety outcomes in patients treated with CW was summarized.

## Results

### Search results

**Fig. 1:**
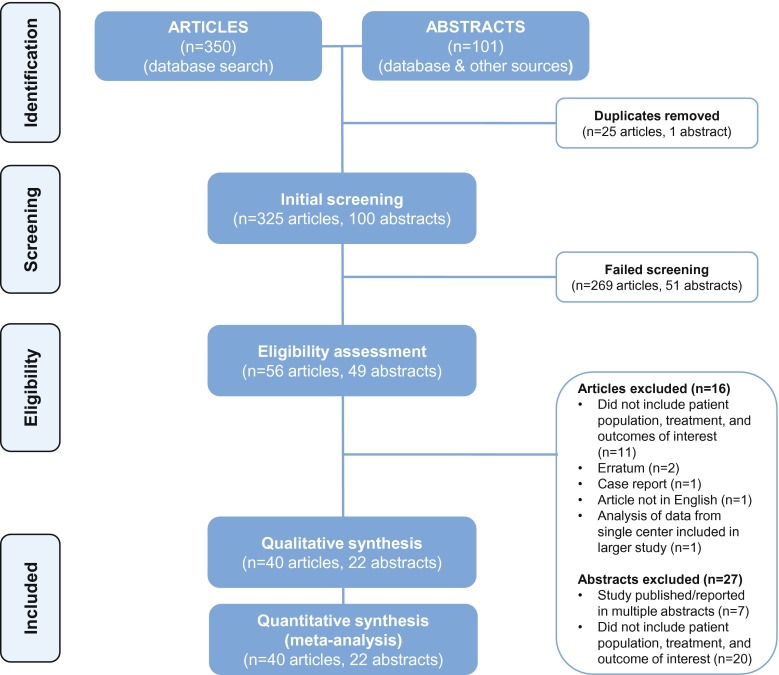
Flow diagram

The initial PubMed search retrieved 350 possible references. Twenty-five duplicate articles were excluded. Based on initial screening of the article abstracts, 269 were excluded (100 were reviews or editorials; 83 did not evaluate CW; 56 were preclinical or phase 1 studies; 22 were individual case reports or small case series; 3 did not evaluate patients with HGG; 3 were pediatric studies; and 2 were secondary or subgroup analyses of published studies). The full text of the remaining 56 references were obtained and reviewed. Sixteen of these references were excluded from the analyses, leaving 40 published reports that were included in the survival or safety analyses. The search of the abstract database identified 22 abstracts for analysis (Fig. 1). Thus, a total of 62 publications were included (Table 1) [6, 11–13, 18–75].

**Table 1:** Characteristics of published studies included in analysis

Study	Design	Diagnosis: new/or recurrent	Grade 3, 4, or both	CW treatment regimen	Other treatment	CW no. of patients	1-year OS (%)	2-year OS (%)
Affronti et al. [18]	RCS, cohort, SC	New	4	Surgery + CW + RT + TMZ + multiagent rotational chemo	Surgery + RT + TMZ + multiagent rotational chemo	36	81	47
Anderson and Thomson (abstr) [19]	RCS, SC	Both	Both	Surgery + CW	n/a	11	Not reported	Not reported
Aoki et al. [20]	Phase 1/2	Both	Both	Surgery + CW + RT + TMZ ± INF-B	n/a	New diagnosis, grade 3 or 4: 16; new diagnosis: grade 4 only: 9; recurrent, grade 3 or 4: 8; recurrent, grade 4 only: 4	New diagnosis, grade 3 or 4: 100; new diagnosis, grade 4 only: not reported; recurrent, grade 3 or 4: 62.5; recurrent, grade 4 only: 50	New diagnosis, grade 3 or 4: 68.8; new diagnosis, grade 4 only: 44.4; recurrent, grade 3 or 4: 25; recurrent, grade 4 only: not reported
Attenello et al. [21]	RCS, cohort, SC	Both	Both	Surgery + CW	Surgery	Primary resection: 166; revision resection: 122; grade 3: 250; grade 4: 38	Primary, grade 3: 78; primary, grade 4: 57; revision, grade 3: 68; revision, grade 4: 47	Primary, grade 3: 66; primary, grade 4: 20; revision, grade 3: 47; revision, grade 4: 13
Barr and Grundy [22]	RCS, SC	Both	Both	Surgery + CW ± RT ± TMZ	n/a	Primary resection: 59; revision resection: 5	Primary: 61; revision: not reported	Primary: 20; revision: not reported
Bock et al. [23]	RCS, MC	New	4	Surgery + CW + RT + TMZ	n/a	44	58	13
Brem et al. [24]	Phase 1/2	Recurrent	Both	Surgery + CW	n/a	21	38	n/a
Brem et al. [13]	RCT, MC	Recurrent	Both	Surgery + CW ± chemo	Surgery + Placebo ± chemo	110	24	11
Catalán-Uribarrena et al. [25]	Prospective cohort	New	Both	Surgery + CW + RT	Surgery + RT ± TMZ	55	52	11
Chaichana et al. [26]	RCCS	New	4	Surgery + CW ± RT ± TMZ	Surgery ± RT ± TMZ	45	33	9
Damilakis 2011 (abstr) [27]	RCS, SC	New	4	Surgery + CW + RT	n/a	22	Not reported	Not reported
Darakchiev et al. [28]	Phase 1/2	Recurrent	4	Surgery + CW + I-125 (± RT ± chemo)	n/a	34	66	23
De Bonis et al. [29]	RCS, SC	Both	4	Surgery + CW ± RT ± TMZ	Surgery ± RT ± TMZ	New: 19 Recurrent: 28	Not reported	Not reported
Della Puppa et al. [30]	RCS, SC	Both	Both	Surgery + CW + chemo ± RT	n/a	36	Not reported	Not reported
Della Puppa et al. [31]	RCS, SC	Both	Both	Surgery + CW + chemo ± RT	n/a	55	Not reported	Not reported
De’Santi et al. [32]	Case series, SC	Both	4	Surgery + 5-ALA + CW + RT + TMZ	n/a	10	Not reported	Not reported
Desjardins et al. (abstr) [33]	Phase 2	New	4	Surgery + CW + RT + TMZ + Bev	n/a	33	Not reported	Not reported
Dörner et al. [34]	RCS, SC	Both	Both	Surgery + CW	n/a	88	Not reported	Not reported
Duntze et al. [35]	Prospective cohort	New	Both	Surgery + CW + RT + TMZ	n/a	92	70.3	37
Giese et al. [36]	Phase 3 RCT (subgroup analysis)	New	4	Surgery + CW + RT	Surgery + placebo + RT	11	Not reported	Not reported
Gutenberg et al. [37]	RCS	Both	4	Surgery + CW ± RT ± TMZ	n/a	New diagnosis, without TMZ: 13; new diagnosis, with TMZ: 17; recurrent, unmethylated: 11; recurrent, methylated: 6	Without TMZ: 78; with TMZ: 100; recurrent: Not reported	Without TMZ: 0; with TMZ: 38; recurrent: not reported
Ho et al. (abstr) [38]	RCS, SC	Not specified	Not stated	Surgery + CW ± RT ± TMZ	Surgery ± RT ± TMZ	26	Not reported	Not reported
Hoffmann (abstr) [39]	RCS, SC	Both	Both	Surgery + CW ± RT ± TMZ	Not specified	34	Not reported	Not reported
Kleinberg 2004 [40]	RCS, SC	New	Both	Surgery + CW +RT	n/a	39	Not reported	Not reported
Ko et al. [41]	RCS	Recurrent	4	Surgery + CW ± I-125	n/a	CW +I-125: 17; CW alone: 7	CW + I-125: 41; CW alone: 16	CW + I-125: 22; CW alone: 0
Krex et al. (abstr) [42]	RCS, SC	Recurrent	4	Surgery + CW + RT + TMZ	n/a	60	Not reported	Not reported
Kunwar et al. [43]	Phase 3 RCT	Recurrent	4	Surgery + CW	Surgery + CB	93	38	13
Lechapt-Zalcman et al. [44]	Prospective observational MC	New	4	Surgery + CW + RT + TMZ	n/a	111	71.7	34.2
Lemcke et al. [45]	RCS	Not specified	Grade 4	Surgery + CW	Surgery alone	30	Not reported	Not reported
Lopez et al. (abstr) [46]	RCS, SC	Recurrent	4	Surgery + CW ± TMZ	n/a	14	64.3	50
McGirt et al. [47]	RCS, cohort, SC	New	4	Surgery + CW + RT ± TMZ	Surgery + RT + TMZ	With TMZ: 30; no TMZ: 78	With TMZ: 92; no TMZ: 57	With TMZ: 39; no TMZ: 18
McGovern et al. [48]	RCS, SC	Recurrent	Both	Surgery + CW	n/a	33	Not reported	Not reported
Menei et al. [49]	RCS, MC	Both	Both	Surgery + CW ± RT ± TMZ/chemo	n/a	New: 83; recurrent: 80	Not reported	Not reported
Metellus et al. [6]	Prospective cohort	Recurrent	4	Surgery + CW	n/a	22	36.4	Not reported
Metellus et al. (abstr) [50]	Prospective cohort	New	4	Surgery + CW + RT	n/a	29	51	Not reported
Miglierini et al. [51]	RCS, SC	New	4	Surgery + CW + RT + TMZ	n/a	24	78	24
Noël et al. [52]	RCS, SC	New	Both	Surgery + CW + RT + TMZ	Surgery + RT + TMZ	Grade 3 or 4: 28; grade 4 only: 20	Grade 3 or 4: 78.6; grade 4 only: 75	Grade 3 or 4: 40.9; grade 4 only: 38.9
Pan et al. [53]	RCS, SC	New	4	Surgery + CW + RT + TMZ	n/a	21	75	39
Perez Gomez et al. [54]	RCS	New	Both	Surgery + CW + RT + TMZ	n/a	49	60.5	21.3
Qadri et al. (abstr) [55]	RCS, SC	Recurrent	Both	Surgery + CW	n/a	Grade 3 o4 4: 20; grade 4 only: 15	Grade 3 or 4: not stated; grade 4 only: 41	Not reported
Qadri et al. (abstr) [56]	RCS, SC	Recurrent	4	Surgery + CW	n/a	14	Not reported	Not reported
Quinn 2009 [57]	Phase 2, OL, SC	Recurrent	4	Surgery + CW + O-6-BG	n/a	52	47	10
Quiros [58]	Retrospective cohort	New	Both	Surgery + CW + RT + TMZ	Surgery + RT + TMZ	35	Not reported	Not reported
Ranjan et al. [59]	Phase 2	New	4	Surgery + CW + RT + TMZ + Bev	n/a	41	Not reported	Not reported
Rezazadeh et al. [abstr] [60]	Phase 2	New	4	Surgery + CW + RT + TMZ + Bev	n/a	10	Not reported	Not reported
Ryken (abstr) [61]	Prospective cohort	New	4	Surgery + CW + RT + TMZ	n/a	21	Not reported	Not reported
Salmaggi et al. [62]	Phase 2	New	4	Surgery + CW + RT + TMZ	n/a	35	85	30
Salvati et al. [63]	RCS, SC	New	4	Surgery + CW + RT + TMZ	n/a	32	100	Not reported
Samis Zella et al. [64]	RCS	Recurrent	4	Surgery + CW ± TMZ ± other chemo	Surgery ± TMZ ± other chemo	63	Not reported	Not reported
Satilmis et al. (abstr) [65]	RCS, SC	Recurrent	4	Surgery + CW	n/a	71	Not reported	Not reported
Shah et al. [66]	RCS	Both	Both	Surgery + CW ± RT ± TMZ	n/a	177 patients (181 surgeries)	Not reported	Not reported
Silvani et al. (abstr) [67]	Phase 2	New	4	Surgery + CW + RT + TMZ	n/a	35	Not reported	Not reported
Smith et al. [68]	Phase 1/2 prospective, SC	New	4	Surgery + CW + GKS + RT	n/a	27	51	22
Subach et al. [69]	RCS matched cohort, SC	Recurrent	4	Surgery + CW	Surgery	17	0	Not reported
Sumrall et al. (abstr) [70]	Phase 1/2	New	Both	Surgery + CW + RT + TMZ	n/a	Grade 4 only: 43	74	Not reported
Uff et al. (abstr) [71]	RCS, SC	Recurrent	Not stated	Surgery + CW	n/a	30	37	Not reported
Ulmer et al. [72]	RCS	Both	4	Surgery + CW ± RT ± TMZ	n/a	44	32	5
Valtonen et al. [11]	Phase 3 RCT	New	Both	Surgery + CW + RT	Surgery + placebo + RT	Grade 3 or 4: 16; grade 4 only: 11	Grade 3 or 4: 64; grade 4 only: 55	Grade 3 or 4: 32; grade 4 only: 19
Watts et al. [73]	Prospective single-arm	New	4	Surgery + 5-ALA + CW + RT + TMZ	n/a	59	Not reported	Not reported
Westphal et al. [12]	Phase 3 RCT	New	Both	Surgery + CW + RT	Surgery + Placebo + RT	Grade 3 or 4: 120; grade 4 only: 101	Grade 3 or 4: 59.2; grade 4 only: 58	See Westphal 2006
Westphal et al. [74]	Long-term follow-up	New	Both	Surgery + CW + RT	Surgery + placebo + RT	Grade 3 or 4: 120; Grade 4 only: 101	See Westphal 2003	Grade 3 or 4: 15.8; grade 4 only: 10
Zhu et al. (abstr) [75]	RCS, SC	New	4	Surgery + CW	n/a	57	Not reported	29.63

Study	3-year OS (%)	Median survival (months)	Other treatment (N)	1-year OS (%)	2-year OS (%)	3-year OS (%)	Median survival (months)
Affronti et al. [18]	21	22.35	49	69	29	20	18.175
Anderson and Thomson (abstr) [19]	Not reported	Not reported	n/a	n/a	n/a	n/a	n/a
Aoki et al. [20]	Not reported	New diagnosis, grade 3 or 4: not calculable; new diagnosis, grade 4 only: 20.2; recurrent, grade 3 or 4: 12; recurrent, grade 4 only: 8.6	n/a	n/a	n/a	n/a	n/a
Attenello et al. [21]	Primary, grade 3: 58; primary, grade 4: 20; revision, grade 3: 29; revision, grade 4: 8	Primary, grade 3: 57; primary: grade 4: 13.5; revision, grade 3: 23.6; revision, grade 4: 11.3	n/a	n/a	n/a	n/a	n/a
Barr and Grundy [22]	Primary: 10; revision: not reported	Primary: 15.3; revision: 7.5	n/a	n/a	n/a	n/a	n/a
Bock et al. [23]	13	12.7	n/a	n/a	n/a	n/a	n/a
Brem et al. [24]	n/a	11.5	n/a	n/a	n/a	n/a	n/a
Brem et al. [13]	9	7.75	112	20	11	8	5.75
Catalán-Uribarrena et al. [25]	11	13.4	55	43	18	11	11.0
Chaichana et al. [26]	1	8.7	45	9	0	0	5.5
Damilakis 2011 (abstr) [27]	Not reported	Not reported	n/a	n/a	n/a	n/a	n/a
Darakchiev et al. [28]	20	17.25	n/a	n/a	n/a	n/a	n/a
De Bonis et al. [29]	Not reported	New: 14; recurrent: 6	New: 58 Recurrent: 60	Not reported	Not reported	Not reported	New: 11; recurrent:9
Della Puppa et al. [30]	Not reported	Not reported	n/a	n/a	n/a	n/a	n/a
Della Puppa et al. [31]	Not reported	Not reported	n/a	n/a	n/a	n/a	n/a
De’Santi et al. [32]	Not reported	21	n/a	n/a	n/a	n/a	n/a
Desjardins et al. (abstr) [33]	Not reported	Not reported	n/a	n/a	n/a	n/a	n/a
Dörner et al. [34]	Not reported	Not reported	n/a	n/a	n/a	n/a	n/a
Duntze et al. [35]	Not reported	18.8	n/a	n/a	n/a	n/a	n/a
Giese et al. [36]	Not reported	14.7	13	Not reported	Not reported	Not reported	9.5
Gutenberg et al. [37]	Not reported	New diagnosis, without TMZ: 14.7; new diagnosis, with TMZ: 18.9; recurrent, unmethylated: 11.2; recurrent, methylated: 10	n/a	n/a	n/a	n/a	n/a
Ho et al. (abstr) [38]	Not reported	Not reported	42	Not reported	Not reported	Not reported	Not reported
Hoffmann (abstr) [39]	Not reported	19.5	n/a	n/a	n/a	n/a	n/a
Kleinberg 2004 [40]	Not reported	12.8	n/a	n/a	n/a	n/a	n/a
Ko et al. [41]	Not reported	CW + I-125: 11.67; CW alone: 7; CW ± I-125: 11.2	n/a	n/a	n/a	n/a	n/a
Krex et al. (abstr) [42]	Not reported	8.9	n/a	n/a	n/a	n/a	n/a
Kunwar et al. [43]	Not reported	8.8	183	38	13	Not reported	9.1
Lechapt-Zalcman et al. [44]	18.1	17.5	n/a	n/a	n/a	n/a	n/a
Lemcke et al. [45]	Not reported	12.8	58	Not reported	Not reported	Not reported	11.4
Lopez et al. (abstr) [46]	Not reported	26	n/a	n/a	n/a	n/a	n/a
McGirt et al. [47]	With TMZ: 32; no TMZ: 12	With TMZ: 21.3; no TMZ: 12.4	45	Not reported	Not reported	Not reported	14.7
McGovern et al. [48]	Not reported	Not reported	n/a	n/a	n/a	n/a	n/a
Menei et al. [49]	Not reported	New: 17; recurrent: 7	n/a	n/a	n/a	n/a	n/a
Metellus et al. [6]	Not reported	9.9	n/a	n/a	n/a	n/a	n/a
Metellus et al. (abstr) [50]	Not reported	12.6	n/a	n/a	n/a	n/a	n/a
Miglierini et al. [51]	15	19.2	n/a	n/a	n/a	n/a	n/a
Noël et al. [52]	Not reported	Grade 3 or 4: 20.6; grade 4 only: 20.8	Grade 3 or 4: 37; grade 4 only: 16	Grade 3 or 4: 78.4; grade 4 only: 62.5	Grade 3 or 4: 33.3; grade 4 only: 0	Not reported	Grade 3 or 4: 20.8; grade 4 only: 13.8
Pan et al. [53]	24	17	n/a	n/a	n/a	n/a	n/a
Perez Gomez et al. [54]	13.3	15	n/a	n/a	n/a	n/a	n/a
Qadri et al. (abstr) [55]	Not reported	Not reported	n/a	n/a	n/a	n/a	n/a
Qadri et al. (abstr) [56]	Not reported	Not reported	n/a	n/a	n/a	n/a	n/a
Quinn 2009 [57]	5	12.575	n/a	n/a	n/a	n/a	n/a
Quiros [58]	Not reported	20	35	Not reported	Not reported	Not reported	20
Ranjan et al. [59]	Not reported	16.1	n/a	n/a	n/a	n/a	n/a
Rezazadeh et al. [abstr] [60]	Not reported	Not reported	n/a	n/a	n/a	n/a	n/a
Ryken (abstr) [61]	Not reported	18.2	n/a	n/a	n/a	n/a	n/a
Salmaggi et al. [62]	Not reported	17.8	n/a	n/a	n/a	n/a	n/a
Salvati et al. [63]	Not reported	Not reported	n/a	n/a	n/a	n/a	n/a
Samis Zella et al. [64]	Not reported	Not reported	32	Not reported	Not reported	Not reported	Not reported
Satilmis et al. (abstr) [65]	Not reported	Not reported	n/a	n/a	n/a	n/a	n/a
Shah et al. [66]	Not reported	Not reported	n/a	n/a	n/a	n/a	n/a
Silvani et al. (abstr) [67]	Not reported	23	n/a	n/a	n/a	n/a	n/a
Smith et al. [68]	9	12.5	n/a	n/a	n/a	n/a	n/a
Subach et al. [69]	Not reported	3.5	45	50	Not reported	Not reported	13.5
Sumrall et al. (abstr) [70]	12	19.35	n/a	n/a	n/a	n/a	n/a
Uff et al. (abstr) [71]	Not reported	15.25	n/a	n/a	n/a	n/a	n/a
Ulmer et al. [72]	Not reported	10.4 (mean)	n/a	n/a	n/a	n/a	n/a
Valtonen et al. [11]	Grade 3 or 4: 27; grade 4 only: 19	Grade 3 or 4: 14.52; grade 4 only: 13.33	Grade 3 or 4: 16; grade 4 only: 16	Grade 3 or 4: 20; grade 4 only: 20	Grade 3 or 4: 7; grade 4 only: 7	Grade 3 or 4: 7; grade 4 only: 7	Grade 3 or 4: 9.98; grade 4 only: 9.98
Watts et al. [73]	Not reported	Not reported	n/a	n/a	n/a	n/a	n/a
Westphal et al. [12]	See Westphal 2006	Grade 3 or 4: 13.8; grade 4 only: 13.1	Grade 3 or 4: 120; grade 4 only: 106	Grade 3 or 4: 49.6; grade 4 only: 48	See Westphal 2006	See Westphal 2006	Grade 3 or 4: 11.6; grade 4 only: 11.4
Westphal et al. [74]	Grade 3 or 4: 9.2; grade 4 only: 1	See Westphal 2003	Grade 3 or 4: 120; grade 4 only: 106	See Westphal 2003	Grade 3 or 4: 8.3; grade 4 only: 5	Grade 3 or 4: 1.7; Grade 4 only: 0	See Westphal 2003
Zhu et al. (abstr) [75]	Not reported	19.38 (mean)	n/a	n/a	n/a	n/a	n/a

*AA* anaplastic astrocytoma, *AO* anaplastic oligodendroglioma, *CW* carmustine wafer, *GBM* glioblastoma multiforme, *GKS* Gamma Knife surgery, *HGG* high-grade glioma, *MC* multicenter, *O* ^*6*^ *-BG* O^6^-benzylguanine, *OL* open label, *RCCS* retrospective case control study, *RCS* retrospective case series, *RCT* randomized controlled trial, *RT* radiotherapy, *SC* single center, *TMZ* temozolomide

### Publication and population characteristics

The 62 publications in this analysis reported data for 60 separate studies; one study (Westphal 2003 [12]) was reported in 3 publications: the primary study report and 2 follow-up analyses [36, 74]. Thus, in all, 60 different study populations were included. In all analyses, sample sizes were calculated for each variable. The total number of patients treated with CW in 60 studies was 3,162 (mean ± SD sample size = 53 ± 47, range 10–288). The total number of patients treated without CW in 17 studies was 1,736 (mean ± SD sample size = 102 ± 167, range 10–725). A total of 3,071 patients treated with CW and 1,663 patients without CW were evaluated for safety; efficacy populations were 2,637 and 1,685, respectively (all analyses were based on the number of patients with data for a specific outcome). The mean ± SD age of patients in the 25 studies reporting mean age was 55 ± 37 years (range from 33 studies reporting range = 17–83 years).

Thirty-eight studies were retrospective studies, seven studies were prospective observational studies, and fifteen were phase 1/2 through 3 clinical trials and/or randomized controlled trials.

Twenty-eight studies included only newly-diagnosed patients, 16 studies included only recurrent patients, and 14 studies included both newly-diagnosed and recurrent patients, while 2 studies [38, 45]) did not specify. Thirty-three studies included patients with grade 4 tumors only; 25 studies included patients with grade 3 or grade 4 HGG; tumor grade was not stated for 2 studies.

Study treatments (for patients treated with CW) were listed as: Surgery + CW-only in 19 studies, Surgery + CW + other treatment(s) in 28 studies, both Surgery + CW-only, and Surgery + CW + other treatments in 10 studies, and not stated in 2 studies. Radiotherapy was used with CW in 38 (63 %) studies, chemotherapy with TMZ was used with CW in 32 (53 %) studies, and other chemotherapy was used in 9 (15 %) studies.

### Efficacy

#### Overall survival

**Fig. 2:**
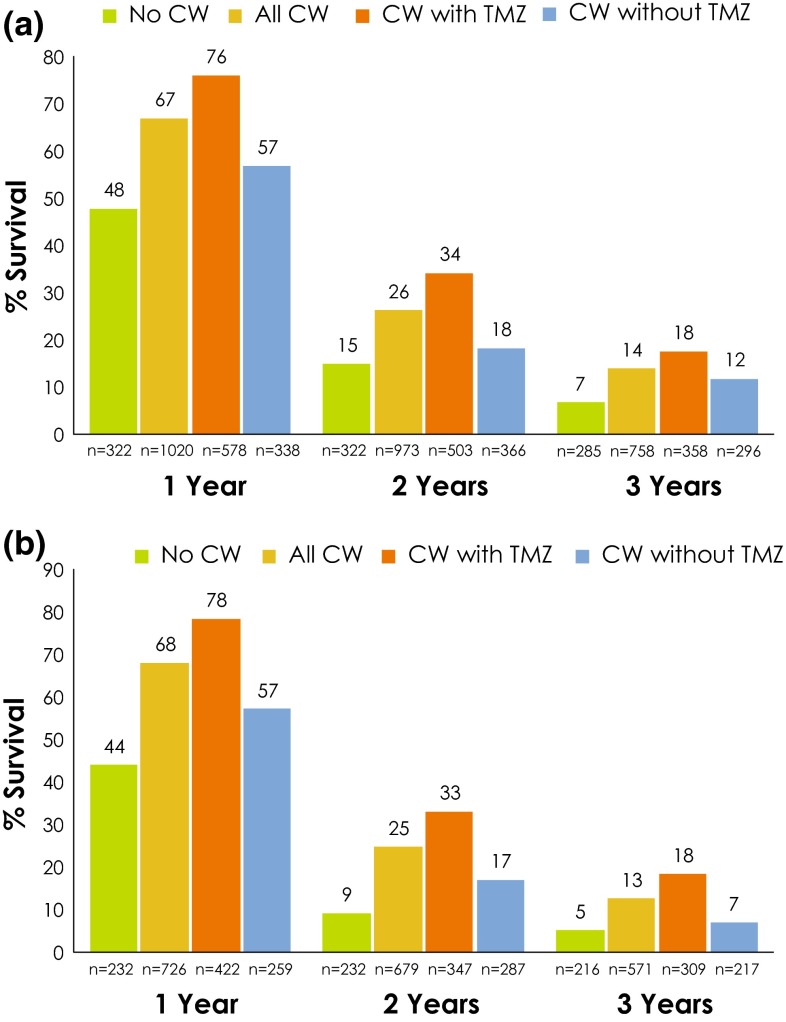
**a**, **b** Overall survival rates in patients with newly-diagnosed HGG **a** grade 3 or 4 and **b** grade 4 only. *CW* carmustine wafer, *HGG* high-grade glioma, *TMZ* temozolomide. If the sum of the n values for CW + TMZ and CW without TMZ subgroups does not equal the n for the All CW subgroup, this is due to the fact that a few studies in the meta-analysis included some patients who received TMZ and some who did not, but results were reported for the entire study group (i.e., not reported separately based on use of TMZ); data from these studies were included in the analysis for All CW, but were excluded from the with/without TMZ analyses

OS was summarized separately for patients with newly-diagnosed HGG and for those with recurrent HGG. Among patients with newly-diagnosed HGG, OS at 1, 2, and 3 years was numerically greater for patients who received treatment with CW compared with those who did not; among those treated with CW, OS was numerically higher for patients who also received TMZ compared with those who did not (Fig. 2a). The same general pattern was observed when data from only patients with grade 4 tumors were analyzed (Fig. 2b).

**Fig. 3:**
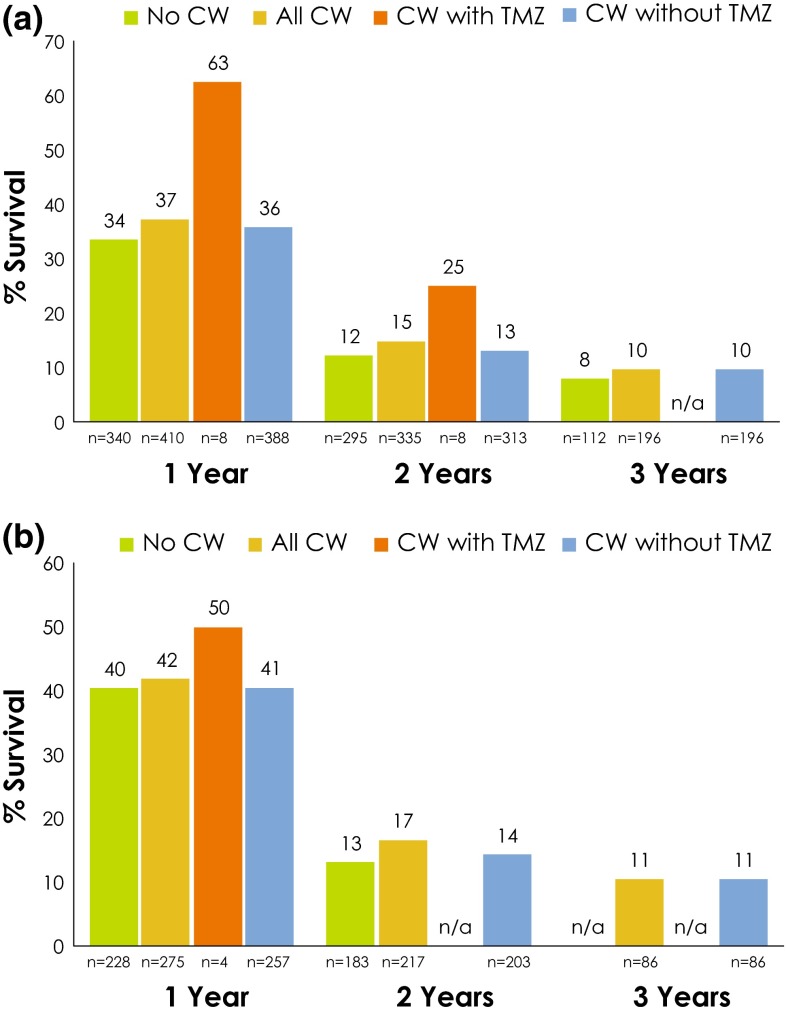
**a**, **b** Overall survival rates in patients with recurrent HGG **a** grade 3 or 4 and **b** grade 4 only. *CW* carmustine wafer, *n/a* not available, *HGG* high-grade glioma, *TMZ* temozolomide. Limited sample size for CW + TMZ. Survival based on time after diagnosis of, or surgery for, recurrent disease. If the sum of the n values for CW + TMZ and CW without TMZ subgroups does not equal the n for the All CW subgroup, this is due to the fact that a few studies in the meta-analysis included some patients who received TMZ and some who did not, but results were reported for the entire study group (i.e., not reported separately based on use of TMZ); data from these studies were included in the analysis for All CW, but were excluded from the with/without TMZ analyses

Survival among patients with recurrence was based on time from diagnosis of surgery for recurrence. Among patients with recurrent HGG, OS at 1, 2, and 3 years was numerically greater for patients treated with CW compared with those who were not; among those treated with CW, OS was numerically higher for patients who also received TMZ compared with those who did not (Fig. 3a). Results were similar in the analysis of data from only patients with grade 4 tumors (Fig. 3b). In both cases, results for patients treated with CW + TMZ should be interpreted with caution, as they are based on a very limited sample of patients.

#### Median survival

**Fig. 4:**
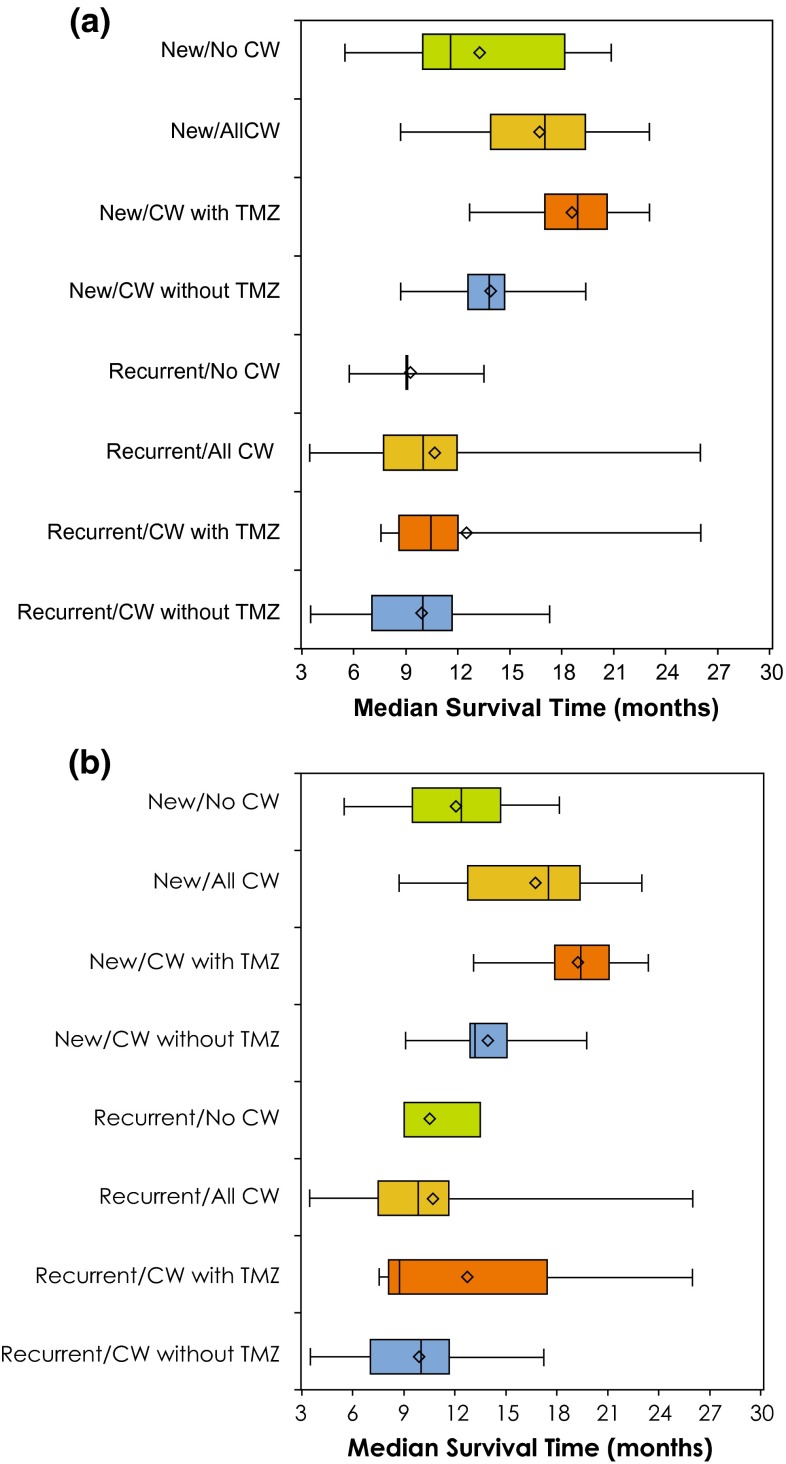
**a**, **b** Median survival among patients with newly-diagnosed or recurrent HGG **a** grade 3 or 4 and **b** grade 4 only; box plots with average (*diamond*), 25th, 50th and 75th percentiles (*lines of the box*) and range (*minimum* and *maximum hash marks*) are also provided for the median survival times (months). Significant effects of treatment (CW vs no CW; *P* = 0.043) and diagnosis (new diagnosis vs recurrence; *P* < 0.001) were detected, with no treatment-by-diagnosis interaction (*P* = 0.620); the effect of TMZ was also significant (*P* < 0.001). *CW* carmustine wafer, *HGG* high-grade glioma, *TMZ* temozolomide. Limited sample size for Recurrent/CW + TMZ. Survival for recurrent diagnosis based on time after diagnosis of, or surgery for, recurrent disease

Median survival for patients with newly-diagnosed and recurrent HGG is shown in Fig. 4a (grade 3 or 4) and Fig. 4b (grade 4 only). Analysis of median survival data showed a significant effect of treatment (median survival was longer with CW than without; *P* = 0.043) and diagnosis (median survival was longer for newly diagnosed HGG than recurrent HGG; *P* < 0.001), with no treatment-by-diagnosis interaction (*P* = 0.620); the effect of TMZ was also significant (*P* < 0.001).

### Safety

There were 28 deaths (28/3,071; 0.91 %) reported as adverse events (AEs) among patients receiving CW, and 34 deaths (34/1,663; 2.0 %) among patients not receiving CW. The single large RCT of only recurrent diagnosed patients of CW vs cintredekin besudotox [43] had all 34 deaths in patients who did not receive CW (34/177 = 19.2 %), and 13 deaths among CW patients (13/92 = 14.1 %, *P* > 0.05). The remaining 15 deaths were reported in 11 studies; most (n = 10) were among newly-diagnosed patients. Not all studies indicated specific AEs resulting in death; among the specific AEs that were cited (for 16 patients treated with CW), pulmonary embolism (n = 3) and stroke (n = 2) were the most common (all others were 1 patient each).

CW removal was performed on 12 patients (12/3,071; 0.39 %) in 5 studies, where 5 patients were recurrent diagnosis patients. In 8 of the 12 patients, the AE term associated with wafer removal was infection at the surgical site.

Repeat surgeries were performed in 83 patients treated with CW (83/3,071; 2.7 %) in 13 studies. The most common AE terms associated with repeat surgeries were surgical site infection (n = 11), hydrocephalus (n = 9), hematoma (n = 8), cysts in resection cavity (n = 7), and wound healing complications (n = 6).

## Discussion

In this meta-analysis of data from patients with newly-diagnosed HGG treated with CW (±other adjuvant treatments), median survival time was 16 months, with 1- and 2-year OS of 67 and 26 %, respectively. Among patients from the same studies who were treated with other modalities, median survival time was 13 months, with 1-year OS of 48 % and 2-year OS of 15 %.

As expected, OS rates were lower (1-year: 37 %; 2-year: 15 % and median survival (approximately 10 months) was shorter among patients treated with CW with recurrent disease relative to those with newly-diagnosed disease. The median survival times reported here are slightly longer than those reported in the prescribing information for CW (13.8 and 7.4 months for new and recurrent glioma, respectively) [10], which are based on 2 phase 3 RCTs [12, 13]. This difference may be due in part to the inclusion of TMZ and other adjuvant treatments or advances in surgical resection techniques, among other factors.

The majority studies included in this analysis enrolled patients with grade 4 gliomas; several studies also included patients with grade 3 gliomas, although outcomes in those studies were not always reported separately by tumor grade. In our analysis, survival outcomes among the subset of patients treated with CW with grade 4 HGG were generally similar to those among all patients (i.e., patients with grade 3 or 4 HGG).

In this analysis, survival was generally improved among patients treated with CW who also received TMZ than among those who received CW without TMZ. This is not unexpected, considering the complementary mechanisms of action of CW and TMZ, as has previously been reviewed [76]. However, the sample sizes for these subgroups were limited. In addition, only a few of the studies that evaluated CW with TMZ accounted for MGMT promoter status [37, 44, 52, 62, 70]. In light of evidence that MGMT promoter status may be a significant predictor of survival in patients treated with CW or TMZ [6, 7], this further limits the ability to draw definitive conclusions from these data with regard to potential treatment-related differences in survival benefit. An extensive exploration of molecular mechanisms and optimization of HGG treatment in an era of personalized medicine is beyond the scope of this discussion. However, we believe that studies will need to be conducted further evaluating the role of CW for use as part of a multi-modal approach with the current “standard of care” of newly-diagnosed GBM, i.e., radiotherapy plus TMZ, followed by monthly adjuvant TMZ, as well as with the many new emerging targeted therapies, including drugs such as bevacizumab. Also in light of the newly-presented data this past year regarding the upfront glioblastoma multiforme bevacizumab studies (RTOG 0825 [77], AVAglio [78]), the utility of CW in HGG may need to be revisited: given the heterogeneity of HGG at a tumor biology level, it may seem prudent to treat these tumors in a multimodality approach fashion and utilize CW with TMZ and radiotherapy in the appropriate patients in the upfront setting.

Among patients treated with CW, the incidence of death reported as an AE was 0.9, <1 % of patients required wafer removal, and approximately 3 % required repeat surgery. The AEs/complications associated with these events were generally consistent with the known safety profile of CW (e.g., surgical site infection, hydrocephalus, wound healing abnormalities, etc.) [10, 14]. Good surgical practice should include proper and careful technique toward ensuring a water-tight dural closure, thus lowering the risk of known AEs.

Two meta-analyses and several reviews have been published that summarized efficacy and safety data from studies of CW [16, 17, 79–83]. In the most recent Cochrane review of CW, Hart and colleagues reported significantly increased survival with CW relative to placebo in primary disease (HR 0.65, 95 % CI 0.48–0.86, *P* = 0.003), and a non-significant difference in recurrence (HR 0.83, 95 % CI 0.62–1.10, *P* = 0.2). Consistent with the aims of that meta-analysis, estimates of survival times were not calculated and data were largely limited to those from RCTs. A recent meta-analysis based on 19 studies that included newly-diagnosed patients with glioblastoma who were treated with CW found a median survival time of 16.2 months [84]. This is consistent with our finding of 16.4 months for the same type of patients. A number of systematic reviews have been published that included data from multiple studies of various designs [16, 17, 79–82]; however, again, estimates of survival times were not calculated, as these reviews summarized individual study data without further analysis. The lack of similarly designed analyses in the literature therefore limits our ability to compare our results with many published reviews.

In contrast to previous reports, with the current meta-analysis we sought to better characterize outcomes with CW using data from as many studies as possible, to aid clinicians in making treatment recommendations and to assist researchers in developing more inclusive clinical trial designs by providing the most comprehensive and reliable survival-related dataset. As such, the inclusion criteria we used in selecting studies were less restrictive than those of more traditional meta-analyses. Thus, the heterogeneity of the included studies in terms of study design, patient characteristics, and study treatments (variability was present not only between studies but within individual studies), must be noted as a limitation. Because our analysis did not control for the potential effects of these variables, which can have an impact on survival outcomes, these factors should be kept in mind when considering the results. In addition, we did not systematically assess each study for potential bias. The nature of the majority of studies (retrospective, single-arm) largely eliminates bias in terms of favoring one treatment over another. However, there is a degree of selection bias inherent in the patient populations studied; that is, patients who are candidates for CW treatment generally have better performance status and have accessible tumors that can be almost completely resected, and therefore a better prognosis than patients who are not candidates for CW treatment.

In this comprehensive review of the literature on CW and meta-analysis of published survival data, we attempted to summarize the cumulative data of numerous studies that have been reported over the past 18 years. Our results highlight benefits in survival of patients in the CW arms versus patients who did not receive CW. There was significant effect of CW treatment on median survival (*P* = 0.043), with higher OS rates for patients with new or recurrent HGG within the cohort treated with CW.

CW is an FDA-approved treatment modality for all newly-diagnosed HGGs, including GBM, anaplastic astrocytoma, anaplastic oligodendroglioma, and anaplastic oligoastrocytoma, as well as for recurrent GBM. It is also now an accepted form of therapy for newly-diagnosed and recurrent HGG in the most recent NCCN guidelines for CNS tumors [2]. Traditionally, the use of CW has precluded patients from accrual to many clinical trials. One frequently cited reason for exclusion is the potential ambiguity that the presence of the wafers have on the assessment of treatment response (or lack thereof) on follow-up MRI scans. However, it should be considered that following the phase of the treatment involving chemotherapy (in the time between resection and radiotherapy), an inflammatory response occurs, which may also contribute to the effect. In the current era of new biologicals entering clinical trials, the combination of an active inflammatory milieu together with an empowered immune system (e.g., dendritic cells, immune checkpoint modulators) may have positive anti-tumor interactions that will have to be determined in trials. Considering the recent trend toward a greater emphasis on OS (rather than PFS), which is largely independent of imaging measures, it may be helpful to reconsider the notion that CW precludes any trial participation. Another important issue contributing to reluctance to use CW involves the lack of reliable survival data for patients treated with CW, which might lead to confusion during the statistical analysis of the survival data of patients in a given trial. With the publication of this new, comprehensive dataset regarding the survival of more than 3,000 patients treated with CW, it should be easier to design clinical trials that can include patients who had received CW. In addition, statisticians will now have more reliable median survival times, 1-year survival rates, and 2-year survival rates to use for the analysis of protocols that will allow accrual of these patients.

## Abbreviations

AA: Anaplastic astrocytoma
AE: Adverse event
AO: Anaplastic oligodendroglioma
CW: Carmustine wafers
GBM: Glioblastoma multiforme
GKS: Gamma Knife surgery
HGG: High-grade glioma
MC: Multicenter
O^6^-BG: O^6^-benzylguanine
OS: Overall survival
RCCS: Retrospective case control study
RCS: Retrospective case series
RCT: Randomized controlled trial
RT: Radiotherapy
SC: Single center
SSI: Surgical site infection
TMZ: Temozolomide
